# Identification of a potential tumor suppressor gene, *UBL3*, in non-small cell lung cancer

**DOI:** 10.20892/j.issn.2095-3941.2019.0279

**Published:** 2020-02-15

**Authors:** Xinchun Zhao, Zhou Yongchun, Hu Qian, Gao Sanhui, Liu Jie, Yu Hong, Zhang Yanfei, Wang Guizhen, Huang Yunchao, Zhou Guangbiao

**Affiliations:** ^1^School of Life Sciences, University of Science and Technology of China, Hefei 230026, China; ^2^State Key Laboratory of Molecular Oncology, National Cancer Center, National Clinical Research Center for Cancer, Cancer Hospital, Chinese Academy of Medical Sciences and Peking Union Medical College, Beijing 100021, China; ^3^State Key Laboratory of Membrane Biology, Institute of Zoology, Chinese Academy of Sciences, Beijing 100101, China; ^4^Department of Thoracic Surgery, the Third Affiliated Hospital of Kunming Medical University, Kunming 650106, China; ^5^School of Chinese Materia Medica, Beijing University of Chinese Medicine, Beijing 100029, China

**Keywords:** Lung cancer, ubiquitin pathway genes, UBL3, tumor suppressor, prognosis

## Abstract

**Objective:** Oncogenes have been shown to be drivers of non-small cell lung cancer (NSCLC), yet the tumor suppressing genes involved in lung carcinogenesis remain to be systematically investigated. This study aimed to identify tumor suppressing ubiquitin pathway genes (UPGs) that were critical to lung tumorigenesis.

**Methods:** The 696 UPGs were silenced by an siRNA screening in NSCLC cells; the potential tumor suppressing UPGs were analyzed, and their clinical significance was investigated.

**Results:** We reported that silencing of 11 UPGs resulted in enhanced proliferation of NSCLC cells, and four UPGs (*UBL3*, *TRIM22*, *UBE2G2*, and *MARCH1*) were significantly downregulated in tumor samples compared to that in normal lung tissues and their expression levels were positively associated with overall survival (OS) of NSCLC patients. Among these genes, *UBL3* was the most significant one. *UBL3* expression was decreased in tumor samples compared to that in paired normal lung tissues in 59/86 (68.6%) NSCLCs, was correlated with TNM stage and sex of NSCLC patients, and was significantly higher in non-smoking patients than in smoking patients. Silencing UBL3 accelerated cell proliferation and ectopic expression of UBL3 suppressed NSCLC *in vitro* and *in vivo*.

**Conclusions:** These results showed that UBL3 represented a tumor suppressor in NSCLC and may have potential for use in therapeutics and for the prediction of clinical outcome of patients.

## Introduction

Lung cancer is the most common cause of cancer-related mortality, which kills 1.8 million people worldwide each year^1^. Lung cancer comprises small cell lung cancer (SCLC) and non-small cell lung cancer (NSCLC). NSCLC divides into large cell carcinoma, lung adenocarcinoma (LUAD), and lung squamous cell carcinoma (LUSC)^2^. Comprehensive sequencing efforts over the past decade have confirmed that lung cancer is a disease of the genome^3–5^. Abnormalities in oncogenes and tumor suppressors act as drivers to initiate the onset and progression of NSCLCs. However, druggable driver molecules are found in only a minority of patients; hence, efforts should be made to uncover the driver genes in the majority of lung cancers^2^.

The ubiquitin (Ub)-dependent modification system is one of the protein degradation systems within the cell and is involved in a variety of cellular processes. In this process, Ub is activated by a Ub-activating enzyme, E1, and transferred to a Ub-conjugating enzyme, E2, which attaches Ub to a target protein through the ε-amino group of a lysine residue with the help of a Ub-protein ligase, E3. The ubiquitinated target protein is then recognized and degraded by the 26S proteasome^6^. Ub-like proteins (UBLs) usually have a UBL domain and have been reported to act as post-translational modifiers. The modification of proteins by UBLs proceeds through an enzyme cascade similar to that used by Ub. As most UBLs have far fewer known substrates than are known for Ub, they require a more limited number of E2-type conjugating enzymes and E3-type ligases^7^. Small Ub-like modifier (SUMO)^8^ and neuronal precursor cell-expressed developmentally downregulated protein-8 (Nedd8)^9^ are two well-known UBLs and have been reported to play important roles in cancer. Both SUMO and Nedd8 are two well-studied UBLs: SUMO is involved in several cellular activities, including cell cycle control, nuclear transport and responses to viral infections, and Nedd8 functions in the regulation of E3 ubiquitin-protein ligases. These two UBLs are implicated in tumorigenesis^8,9^. Some oncogenic Ub-pathway genes (UPGs) have been reported in NSCLCs^10–12^, but the roles of tumor suppressing UPGs in this deadly disease remain to be elucidated. In this study, we conducted a systematic silencing of the E1, E2, E3, and deubiquitinases in NSCLC cells to identify tumor suppressing UPGs that were crucial for lung cancer cell proliferation.

## Materials and methods

### Patient samples

The study was approved by the local Research Ethics Committees of all the participating sites; all lung cancer samples were collected with informed consent. The diagnosis of lung cancer was confirmed by at least two pathologists. Tissue samples were obtained at the time of surgery and quickly frozen in liquid nitrogen. Tumor samples contained a tumor cellularity of greater than 60%, and the matched control samples contained no tumor cells.

### Cell culture and siRNA library

The NSCLC cell lines A549, H1975, and H460 were obtained from the American Type Culture Collection (ATCC; Manassas, VA, USA). These cells were cultured in Dulbecco’s Modified Eagle’s Medium (DMEM) or Roswell Park Memorial Institute (RPMI) 1640 medium supplemented with 10% fetal bovine serum (FBS), 100 U/mL penicillin, and 100 µg/mL streptomycin (GIBCO BRL, Grand Island, NY, USA). Human siGENOME SMARTpool siRNA libraries, containing 696 UPGs (Catalog numbers G-004705-05, G-005615-05, G-005625-05, G-005635-05), were purchased from Thermo Scientific Dharmacon (Lafayette, CO, USA). The small interfering RNAs (siRNAs) were transfected into cells using DharmFECT transfection reagent (#T-2001-01), and cell viability was assessed by CellTiter-Glo Reagent (Promega, Fitchburg, WI, USA) according to the manufacturer’s instructions. Each SMARTpool experiment was performed in duplicate. The Z-score was calculated as follows: *z* = (*x* − *m*)/*s*, where *x* is the raw score to be standardized, *m* is the mean of the plate, and *s* is the standard deviation (SD) of the plate^13^. The Z-score of a gene reflects its requirement for cell proliferation, and a Z-score of ≤ −2 indicates that the gene is required for cell proliferation; silencing of the gene significantly inhibits cell proliferation. However, a Z score of ≥ 2 indicates that the gene inhibits lung cancer cell proliferation and that inhibition of the gene significantly promotes cell proliferation.

### Cell cycle

To assess the cell cycle distribution, cells were harvested and washed in phosphate-buffered saline (PBS), fixed in 70% ethanol and incubated at 4 °C overnight. Cells were centrifuged and washed with PBS containing 1% FBS, followed by treatment with 1% RNase A for 15 min at 37 °C and staining with 50 µg/mL propidium iodide (Sigma-Aldrich, St. Louis, MO, USA). The fluorescence intensity was measured by flow cytometry (FACSVantage Diva, BD Biosciences, San Jose, CA, USA).

### Antibodies

The antibodies used included mouse anti-β-actin (#A5441; Sigma-Aldrich; 1:5000 for Western blot), mouse anti-HA (#AE008; ABclonal, Cambridge, MA, USA; 1:2000 for Western blot), rabbit anti-ubiquitin-like 3 (UBL3) (#A4028; Abclonal; 1:1000 for Western blot and 1:100 for immunohistochemistry (IHC)), rabbit anti-Ki67 (#ab15580; Abcam, Cambridge, MA, USA; 1:400 for IHC), rabbit anti-p27 (#sc-528; Santa Cruz Biotechnology, Santa Cruz, CA, USA; 1:1000 for Western blot), rabbit anti-Cyclin D1 (#2922; Cell Signaling Technology, Beverly, MA, USA; 1:1000 for Western blot), and mouse anti-Cyclin E (#4129; Cell Signaling Technology; 1:1000 for Western blot).

### The siRNA and plasmid transfection

The siRNA was purchased from GenePharma Co., Ltd. (Shanghai, China), and the sequences were as follows: UUCUCCGAACGUGUCACGUTT (siNC); GAGAGUAAUUGUUGUGUAA (#si*UBL3*-1); and CGGCGGAUAUGAUAAAUUU (#si*UBL3*-2). The HA-*UBL3* vector was constructed based on the pCS2 plasmid. Cells were transfected with siRNA or plasmids using Lipofectamine 3000 reagent (Invitrogen, Carlsbad, CA, USA).

### Lentivirus-mediated cell transfection and transduction

For lentiviral particle production, pCDH-*UBL3* constructs were co-transfected with psPAX2 and pMD2G into HEK293T cells. The culture medium was replaced with fresh medium after 6 h, and supernatant was harvested 48 h and 72 h post-transfection. A549-luciferase cells were infected with viral particles in the presence of 8 µg/mL Polybrene^®^. One week after infection, cells were sorted by flow cytometry.

### Western blot

Cells were lysed in RIPA buffer supplemented with protease inhibitor cocktail (Sigma-Aldrich). Proteins (20 µg) were subjected to 10%–15% sodium dodecyl sulfate polyacrylamide gel electrophoresis (SDS-PAGE), electrophoresed, and transferred to nitrocellulose membranes. After blocking with 5% nonfat milk in Tris-buffered saline, membranes were washed and incubated with the indicated primary and secondary antibodies. Immunoreactions were detected using a Luminescence Image Analyzer LAS 4000 (GE, Fairfield, CO, USA).

### Animal studies

Animal studies were approved by the Institutional Review Board of the Chinese Academy of Medical Sciences Cancer Institute and Hospital, with the approval ID of NCC2019A188. The methods were performed in accordance with the approved guidelines. Six-week-old SCID-beige mice were maintained under specific pathogen-free (SPF) conditions. The mice were numbered, injected into the lateral tail veins with A549-luciferase cells (5 × 10^5^) stably expressing pCDH-*UBL3* or pCDH-control plasmid, and randomized into groups (*n* = 13 per group). After 45 days, tumors were monitored with the IVIS Spectrum Imaging System (Caliper Life Sciences; Hopkinton, MA, USA). For IHC, sections were fixed in formalin and embedded in paraffin, incubated with primary antibodies overnight and incubated with the indicated secondary antibodies. Immunoreactions were detected using 3,3′-diaminobenzidine (DAB, Zhongshan Golden Bridge Biotechnology Co., Ltd., Beijing, China) and hematoxylin.

## Online data availability

The Oncomine^14^ cancer microarray database (www.oncomine.org), including the Hou Lung, Selamat Lung, Su Lung, StearmanLung, Landi Lung, Okayama Lung, Lee lung, Kuner Lung, and Zhu Lung datasets, with the accession codes GSE19188, GSE32867, GSE7670, GSE2514, GSE10072, GSE31210, GSE8894, GSE10245, and GSE14814, respectively, were used. The transcriptome data and clinical data of 517 patients with LUAD and 501 patients with LUSC were downloaded from the Cancer Genomics Hub (https://xenabrowser.net/datapages/). TCGA database was accessed using the accession code phs000178. The online survival analysis software containing the microarray data of patients with NSCLC^15^ (http://kmplot.com/analysis/index.php?p=service&cancer=lung) was used. The correlation of *UBL3* expression with OS was evaluated by entering *UBL3* into the database and analyzed by setting different clinical parameters, and the Kaplan-Meier survival plots and log-rank *P* values were obtained from the webpage. The baseline demographic characteristics of the patients are shown by Gyorffy et al.^15^.

### Statistical analysis

All statistical analyses were conducted using GraphPad Prism 7 (GraphPad Software, La Jolla, CA, USA). All experiments were repeated at least three times, and the data were presented as the mean ± SD unless otherwise noted. Differences between groups of data were evaluated for significance using Student’s *t*-test for unpaired data or one-way analysis of variance (ANOVA). The survival curve for each group was estimated by the Kaplan-Meier method and log-rank test. *P* values less than 0.05 indicated statistical significance.

## Results

### Identification of 11 tumor suppressing UPGs in NSCLCs

**Figure 1:**
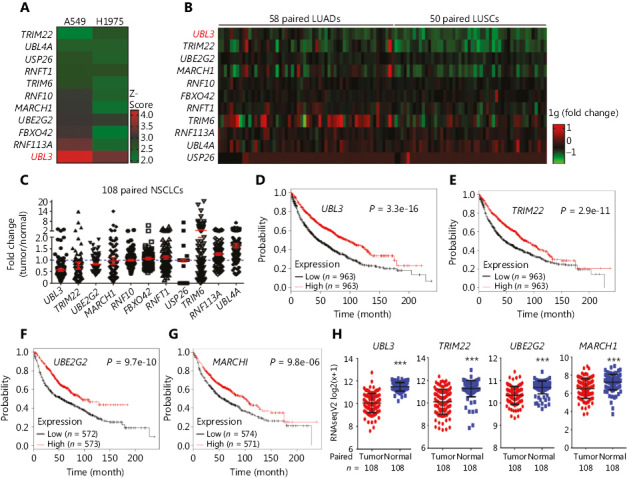
Identification of *UBL3* as a potential tumor suppressor in non-small cell lung cancer (NSCLC) cells. (A) Heat map showing 11 candidates with Z-scores ≥ 2 in both A549 and H1975 cells. (B, C) Heat map and scatter plot of the expression of the 11 candidates in 58 paired lung adenocarcinoma (LUAD) tissues and 50 paired lung squamous cell carcinoma (LUSC) tissues. The fold change indicates the tumor/normal ratio. (D–G) The association between the expression levels of the four genes and the prognosis of patients as determined by online survival analysis software. (H) The expression levels of the four genes in TCGA datasets. ***P < 0.0001.

To systematically identify UPGs that are crucial for lung cancer cell proliferation, we conducted a systematic silencing of the UPGs in NSCLC cell lines. The 696 siRNAs for UPGs in the Dharmacon human siGENOME SMARTpool library were transfected into the NSCLC lines A549 and H1975. Cell viability was measured 72 h after transfection, and robust Z-scores from duplicate experiments for each SMARTpool were determined^13^. While genes with a Z score ≤ −2 have been analyzed in other work^16^, UPGs with a Z-score ≥ 2 (indicating that cell proliferation was increased when the gene was silenced) were assessed here and 11 UPGs were identified (**Figure 1A** and **Supplementary Figure S1**). These included *UBL3*, *USP26*, *UBL4*, *UBE2G2*, *FBXO42*, *RNF10*, *TRIM6*, *LOC51136*, *RNF113A*, *MARCH1*, and *TRIM22*.

### Unveiling UBL3 in NSCLCs

We analyzed the expression levels of the above UPGs in The Cancer Genome Atlas (TCGA) datasets containing 58 paired LUADs and 50 paired LUSCs (**Supplementary Table S1**; **Figure 1B and 1C**), and reported that the expressions of five genes (*UBL3*, *TRIM22*, *UBE2G2*, *MARCH1*, and *RNF10*) were downregulated in these NSCLCs. The association between the expression levels of these five genes and the OS of the patients was analyzed using online survival analysis software^15^ (http://kmplot.com/analysis/index.php?p=service&cancer=lung). We found that the expression of four genes, *UBL3*, *TRIM22*, *UBE2G2*, and *MARCH1*, was positively associated with the OS of the patients, and the median OS of patients with high levels of these genes was significantly longer than that of patients with low levels of these genes (**Figure 1D–G**). In TCGA datasets, the expression levels of the four genes in tumors were lower than those in normal tissues at the mRNA level (**Figure 1H**), and UBL3 represented the most significant one (**Figure 1H**). Therefore, *UBL3* was selected for further investigation.

### The expression of UBL3 in NSCLC

**Figure 2:**
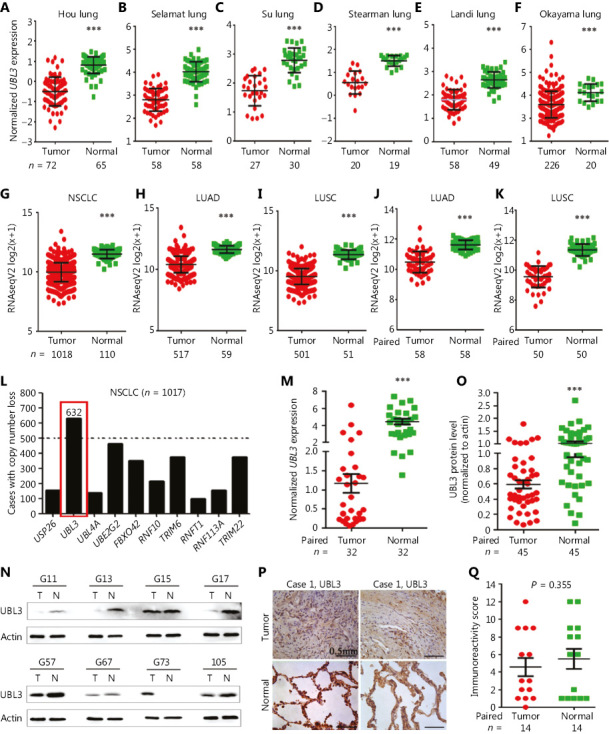
The expression of UBL3 in patients with NSCLC in the Oncomine and TCGA datasets. The expression of *UBL3* in the Hou Lung (A), Selamat Lung (B), Su Lung (C), Stearman lung (D), Landi Lung (E), and Okayama lung (F) Oncomine datasets and the NSCLC (G), lung adenocarcinoma (LUAD) (H), and lung squamous cell carcinoma (LUSC) (I) of TCGA datasets is shown. (J, K) *UBL3* expression levels in tumor tissues and the counterpart adjacent normal lung tissues from the TCGA LUAD (J) and LUSC (K) datasets. (L) Copy number loss analysis of 10 candidates in the TCGA database. (M–Q) The expression of *UBL3* was tested by quantitative reverse transcription polymerase chain reaction (qRT-PCR) (M), Western blot (N, O), and immunohistochemistry (P, Q) assays of lung cancer patients of our cohort. Size bar, 0.5 mm. The densitometry analysis of the Western blot results (O) and the immunoreactivity score of UBL3 were calculated (Q). *P* values, Student’s *t-*test. ***P < 0.0001.

To evaluate the expression of *UBL3* in NSCLC, the Oncomine Cancer Microarray Database^14^ (www.oncomine.org) was explored. In the Hou Lung^17^, Selamat Lung^18^, Su lung^19^, Stearman Lung^20^, Landi Lung^21^, and Okayama Lung^22^ datasets, the *UBL3* mRNA levels were significantly reduced in tumor tissues compared to those in the counterpart normal lung tissues (**Figure 2A–F**). We investigated *UBL3* RNA levels in TCGA dataset containing 1, 018 NSCLC tumor samples and 110 normal lung specimens and found significantly reduced expression of *UBL3* in NSCLC tumor samples compared to that in normal lung tissue samples (**Figure 2G**). In both lung adenocarcinoma (LUAD, *n* = 517) and lung squamous cell carcinoma (LUSC, *n* = 501), the expression of *UBL3* was significantly higher in normal tissues than in tumor lung samples (**Figure 2H and 2I**; *P* < 0.0001). In paired tumor adjacent samples from TCGA datasets, *UBL3* expression in tumor tissues was lower than that in the adjacent lung samples (**Figure 2J** and **2K**, *P* < 0.0001). In addition, the *UBL3* copy number loss was found in 632 (62.1%) of 1,017 TCGA patients (**Figure 2L**).

We tested the expression of *UBL3* in 86 NSCLCs (**Table 1**) by quantitative reverse transcription polymerase chain reaction (qRT-PCR) (**Figure 2M**), Western blot analysis (**Figure 2N and 2O**), and immunohistochemistry (IHC) (**Figure 2P and 2Q**), and observed a decrease in *UBL3* expression in tumor tissues of 59/86 (68.6%) patients, demonstrating the suppressed expression of *UBL3* in NSCLCs.

### UBL3 expression and prognosis of smoker patients with NSCLC

**Figure 3:**
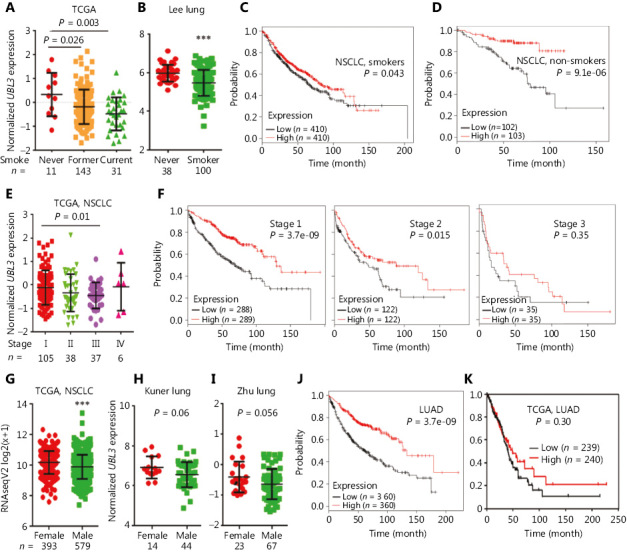
The association between UBL3 expression and clinical characteristics. The expression of *UBL3* in smoking and non-smoking patients with NSCLC in TCGA dataset (A) and Oncomine Lee Lung dataset (B). *UBL3* expression and prognosis of smoking (C) and non-smoking (D) patients with NSCLC as determined by online survival analysis software. (E) The expression of *UBL3* in patients with NSCLC at different stages. (F) Overall survival (OS) of patients with NSCLCs at different stages with high or low expression of *UBL3*. (G) Expression of *UBL3* in female and male patients with NSCLC in TCGA (G) and the Oncomine datasets Kuner Lung (H) and Zhu Lung (I). The Kaplan-Meier survival curve of LUAD patients with high or low levels of *UBL3* expression as determined by online survival analysis software (J) and TCGA database analysis (K). ***P < 0.0001.

The association between the *UBL3* expression levels and tobacco smoking status of the patients was explored in TCGA and Oncomine databases. In TCGA datasets, the expression of *UBL3* was significantly higher in non-smoking NSCLC patients than in former and current smoking NSCLCs (**Figure 3A**). In the Oncomine Lee Lung dataset^23^, *UBL3* in non-smoking patients was also higher than in smoking patients with NSCLC (**Figure 3B**). We analyzed the potential association between *UBL3* expression and patient prognosis using the online survival analysis software. The results showed that smoking patients with NSCLC with high levels of *UBL3* had a favorable prognosis, but non-smoking patients with high levels of *UBL3* had much better prognosis (**Figure 3C and 3D**). These results suggest that the expression of *UBL3* in NSCLC may be modulated by tobacco smoke and related carcinogens and thus involved in lung carcinogenesis. This possibility could be further investigated in the future.

### The association between UBL3 expression and clinical characteristics

We also explored the association between *UBL3* expression levels and TNM stage in patients in TCGA database, and the data showed that the expression of *UBL3* was significantly higher in stage I NSCLCs than in stage II and III NSCLCs (**Figure 3E**). The prognostic significance of *UBL3* was further analyzed using stage-matched samples. Among stage I and stage II patients, those with higher expression of *UBL3* had much longer survival time than those with lower levels of *UBL3* (**Figure 3F**). Among patients with stage III NSCLCs (**Figure 3F, right panel**), those with higher *UBL3* had slightly but not statistically significantly shorter OS than those with lower expression of *UBL3.* The expression of *UBL3* was significantly higher in female NSCLC patients than in male NSCLC patients in TCGA dataset (**Figure 3G**). In Oncomine datasets^24,25^, *UBL3* expression in female patients was also higher than in male patients (**Figure 3H and 3I**), although the difference was not statistically significant, and was possibly attributed to less smoking in women than in men. Analysis with online survival analysis software indicated that LUAD patients with lower *UBL3* expression had significantly worse prognosis than those with higher *UBL3* expression (**Figure 3J**). Similarly, in TCGA datasets, LUAD patients with lower *UBL3* expression had poorer prognosis, although the difference was not statistically significant (**Figure 3K**). These data demonstrated that UBL3 might play a role in the pathogenesis of NSCLC.

### Knockdown of UBL3 promotes lung cancer cell proliferation

**Figure 4:**
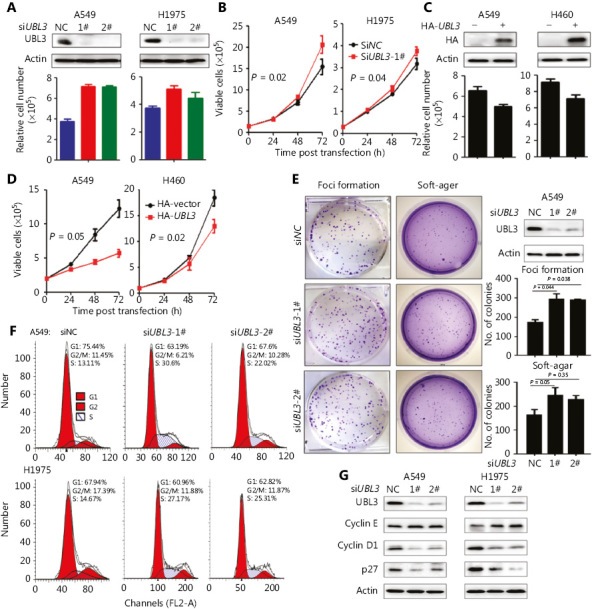
UBL3 inhibits NSCLC cell proliferation. (A) A549 and H1975 cells were transfected with si*UBL3* (1# and 2#) and lysed 72-h later for the detection of UBL3 expression by Western blot assays (upper panel) and assessment of cell viability (lower panel). Error bars, SD. (B) A549 and H1975 cells were transfected with si*UBL3*-1, and cell proliferation was assessed by a Trypan Blue dye exclusion assays. (C, D) A549 and H460 cells were transfected with HA-tagged *UBL3*, and cell proliferation was assessed by a Trypan Blue exclusion assay. (E) Foci formation and soft agar assays of A549 cells transfected with si*UBL3*. Error bars, SD; *P* values, Student’s *t-*test. (F) A549 and H1975 cells were transfected with si*UBL3* and the cell cycle distribution was assessed by propidium iodide staining of DNA followed by flow cytometric analysis. (G) A549 and H1975 cells were transfected with si*UBL3*, lysed 72 h later, and the lysates were subjected to Western blot assay using indicated antibodies.

We found that silencing UBL3 accelerated cell proliferation, with Z-scores of 4.22 and 3.42 in A549 and H1975 cells, respectively (**Figure 1A** and **Supplementary Figure S1**). We verified the effects of siRNA against *UBL3* (si*UBL3*) on lung cancer cells, and found that si*UBL3* treatment resulted in a reduction of UBL3 protein expression and increased viability of A549 and H1975 cells (**Figure 4A**). Knockdown of UBL3 led to a significant increase in cell growth (**Figure 4B**). In contrast, exogenous expression of UBL3 (by transient transfection) significantly inhibited the proliferation (**Figure 4C**) and growth (**Figure 4D**) of cells. We further showed that knockdown of UBL3 promoted the colony-forming activity of A549 cells (**Figure 4E**). Silencing UBL3 in H1975 cells led to cell cycle arrest at the S phase (**Figure 4F**), downregulation of p27, Cyclin D1, and upregulation of Cyclin E (**Figure 4G**), indicating that UBL3 played a role in the control of the replication checkpoint.

### UBL3 inhibits tumor growth *in vivo*

**Figure 5:**
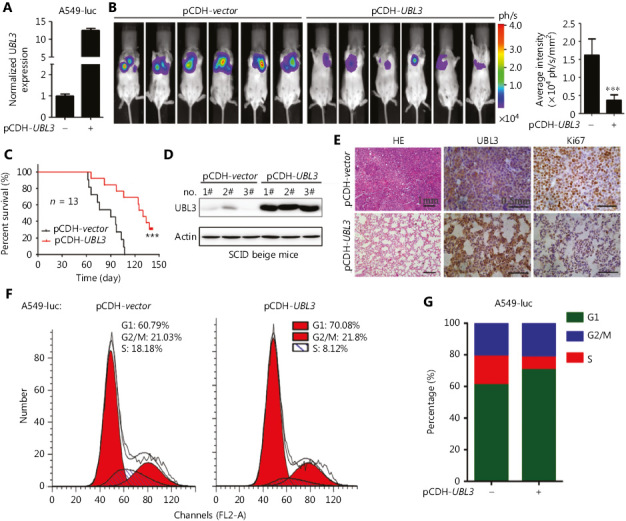
UBL3 suppresses lung cancer *in vivo*. (A) qRT-PCR analysis of UBL3 mRNA levels in pCDH-*UBL3*-expressing A549-luciferase cells. (B) A total of 5 × 10^5^ pCDH-*UBL3*-expressing A549-luciferase cells were injected into SCID-beige mice and the relative luciferase intensity was determined by an IVIS Spectrum system at the indicated time point (B). Error bars, SD; *P* values, Student’s *t-*test. The Kaplan-Meier survival curve of the mice is shown (C). *P* value, log-rank test. Mice were sacrificed, and lung tissues were lysed for Western blot assays using the indicated antibodies (D) or subjected to hematoxylin-eosin (HE) or immunohistochemical staining (E). Scale bar, 1 mm for HE and 0.5 mm for IHC. (F, G) A549-luc cells with pCDH-vector or pCDH-*UBL3* were assessed by propidium iodide staining of DNA followed by flow cytometric analysis (F), and cell cycle distribution was assessed (G).

To investigate the role of UBL3 in tumor growth *in vivo*, the pCDH-*UBL3* plasmid was transfected into A549-luciferase cells (**Figure 5A**), which were then injected into SCID-beige mice via the tail vein (*n* = 13 for each group). To characterize the growth of the xenograft tumors, the luciferase intensity in the mice was measured by an IVIS Spectrum system 45 days after cell inoculation. We found that UBL3 significantly decreased the tumor burden (**Figure 5B**) and extended the life span of the mice (**Figure 5C**). Western blot assays of tumor lysates indicated overexpression of UBL3 *in vivo* (**Figure 5D**). Hematoxylin-eosin (HE) and Ki67 staining of lung sections confirmed the tumor-inhibitory effect of UBL3 (**Figure 5E**). Cell cycle analysis showed that overexpression of UBL3 induced arrest at G1 phase, without accumulation of sub-G1 content (**Figure 5F and 5G**).

## Discussion

In this study, we carried out an siRNA screen for 696 UPGs found in the human genome, and reported 11 candidates that were able to promote cell proliferation when they were silenced in A549 and H1975 cells (**Figure 1**). In TCGA database, the expressions of four potential tumor suppressor genes (*UBL3*, *TRIM22*, *UBE2G2*, and *MARCH1*) were significantly downregulated in tumor samples compared to that in normal lung tissues. Using online survival analysis software^15^, we found that the expression levels of these four genes were positively associated with the OS of patients with NSCLC, with *UBL3* as the most significant one. These results provided rationales for us to further investigate the role of UBL3 in lung carcinogenesis.

UBL3 has been identified in *Drosophila melanogaster*^26^ and is a membrane protein localized by prenylation^27^. UBL3 acts as a post-translational modification factor that regulates protein sorting to small extracellular vesicles (sEVs)^28^. In melanocytic cells, UBL3 may be regulated by MITF^29^. A genome-wide association study demonstrated that UBL3 harbors a susceptibility locus for biliary atresia^30^. Additionally, UBL3 was identified to harbor a novel susceptibility locus for BRCA1/2-negative, high risk breast cancers in Asian women^31^. UBL3 was upregulated in human prostate cancer cells (LNCaP cells) exposed to silvestrol^32^. UBL3 was identified as part of a 7-gene signature (*UBL3*, *FGF3*, *BMI1*, *PDGFRA*, *PTPRF*, *RFC4*, and *NOL7*) for predicting relapse and survival in early-stage patients with cervical carcinoma^33^. Promoter hypermethylation and downregulation were found in *UBL3* in a northeast Indian population with esophageal cancer^34^. An in-frame fusion of MAP3K8-UBL3 was reported as a distinct genetic driver in a unique subgroup of spitzoid neoplasms^35^. Previous studies showed that UBL3 was abnormally expressed in BRCA1/2-negative, high risk breast cancers (BRCAX)^31^, cervical carcinoma^33^, and esophageal cancer^34^. However, the role of UBL3 in lung cancer remains poorly understood. Here, we found that *UBL3* expression was reduced in tumor samples from both TCGA and Oncomine datasets and in 86 NSCLC samples from our laboratory (**Figure 2**, **Table 1**). *UBL3* expression levels were much lower in patients with stage II or III NSCLC than in patients with stage I NSCLC (**Figure 3E**). LUAD patients with higher levels of *UBL3* had more favorable prognosis than those patients with lower levels of *UBL3* (**Figure 3**). We investigated the function of UBL3 in NSCLC cells and demonstrated that silencing of UBL3 promoted, whereas overexpression of UBL3 suppressed, NSCLC cell proliferation *in vitro* and *in vivo* (**Figure 4** and **Figure 5**). These data indicated that UBL3 could be a tumor suppressor in NSCLCs. However, why UBL3 was suppressed in tumor samples and how it suppressed cell proliferation, warrant further investigation.

Tobacco is responsible for about 85% of lung cancer deaths (1.53 million) each year worldwide^1^. Tobacco smoke causes genomic mutations in cancers^36^; promotes cell proliferation; inhibits programmed cell death; facilitates angiogenesis, invasion and metastasis; and enhances tumor-promoting inflammation^37,38^. We found that the expression of *UBL3* was significantly higher in non-smoking than in smoking patients and that non-smoking patients with NSCLC with high levels of *UBL3* had much more favorable prognosis than those who smoked (**Figure 3**). The effects of tobacco smoke on *UBL3* expression need to be investigated in the future.

Collectively, the above results suggested that UBL3 may be involved in lung carcinogenesis by altering the expression of oncoproteins/tumor suppressors, thus affecting critical pathways to promote cell proliferation and disease progression. These possibilities and whether UBL3 could be a therapeutic target for lung cancer warrant further investigation.

## Figures and Tables

**Table 1:** Summary of the baseline demographic characteristics of the 86 patients

Characteristics	Cases, *n*	UBL3-low, *n* (%)	*P*
Total	86	59 (68.6)	
Gender			
Male	48	36 (75)	0.402
Female	20	13 (65)	
Not recorded	18	10 (55.6)	
Age (years)			
< 65	47	33 (70.2)	0.691
≥ 65	20	15 (75)	
Not recorded	19	11 (57.9)	
Smoking status			
Smoker	39	27 (69.2)	0.547
Non-smoker	29	22 (75.9)	
Not recorded	18	10 (55.6)	
Histology			
LUAD	39	29 (74.4)	0.729
LUSC	23	18 (78.3)	
LUAD + LUSC	4	0 (0)	
SCLC	2	2 (100)	
Not recorded	18	10 (55.6)	
TNM Stage			
I-II	36	23 (63.9)	0.150
III-IV	30	24 (80)	
Not recorded	20	12 (60)	

LUAD, lung adenocarcinoma; LUSC, lung squamous cell carcinoma. *P* values were calculated using a two-sided Fisher’s exact test. “UBL3-low” indicates that the levels of UBL3 were lower in tumor tissues than in normal tissues.
