# Supplementary material for: Identification of a potential tumor suppressor gene, UBL3, in non-small cell lung cancer
Source: Cancer Biol Med. 2020 Feb 15;17(1):76–87. doi: 10.20892/j.issn.2095-3941.2019.0279 (PMC7142850; doi:10.20892/j.issn.2095-3941.2019.0279)
Supplement: Supplementary file 1 [file cbm-17-076-s001.pdf]

# Supplementary materials

**Table S1** Summary of the baseline demographic characteristics of the 108 patients with non-small cell lung cancer (NSCLC) in TCGA database

| Characteristics | Cases, *n* | UBL3-low, *n* (%) | *P* |
|---|---|---|---|
| Total number | 108 | 76 (70.4) | |
| Age (years) | | | |
| < 65 | 41 | 26 (63.4) | 0.216 |
| ≥ 65 | 67 | 50 (74.6) | |
| Gender | | | |
| Male | 51 | 36 (70.6) | 0.009 |
| Female | 57 | 26 (45.6) | |
| Smoking status | | | |
| Smoker | 95 | 72 (75.8) | 0.275 |
| Non-smoker | 7 | 4 (57.1) | |
| Not recorded | 6 | 4 (66.7) | |
| Histology | | | |
| LUAD | 58 | 30 (51.7) | 4.86E-06 |
| LUSC | 50 | 46 (92) | |
| TNM stage | | | |
| I-II | 85 | 57 (67.1) | 0.092 |
| III-IV | 21 | 18 (85.7) | |
| Not recorded | 2 | 1 (50) | |
| Anatomic subdivision of lung neoplasm | | | |
| Upper | 63 | 43 (68.3) | 0.619 |
| Lower | 37 | 27 (73) | |
| Middle | 4 | 4 (100) | |
| Not recorded | 4 | 2 (100) | |
| Vital status | | | |
| Living | 56 | 32 (57.1) | 0.002 |
| Dead | 52 | 44 (84.6) | |

*P* values were calculated using a two-sided Fisher's exact test. "UBL3-low" indicates that the levels of UBL3 were lower in tumor tissues than in normal tissues. LUAD, lung adenocarcinoma; LUSC, lung squamous cell carcinoma.

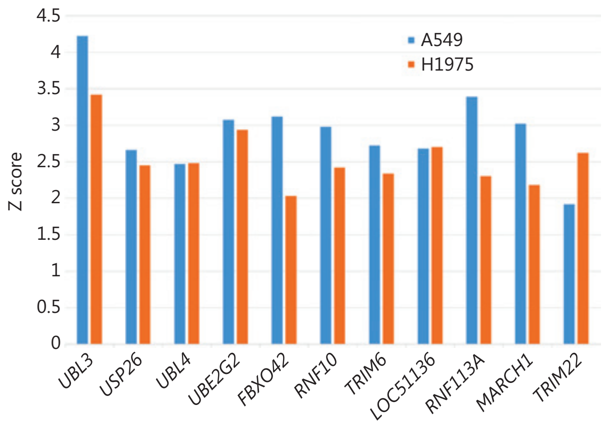


**Figure S1** The 11 UPGs that have a Z score of ≥ 2.
